# Effects of latent infection of *Toxoplasma gondii* strains with different genotypes on mouse behavior and brain transcripts

**DOI:** 10.1186/s13071-025-06819-7

**Published:** 2025-05-26

**Authors:** Bei-Bei Zhou, Hong-Jie Dong, Hang Sun, Xiao-Man Xie, Huan-Huan Xie, Wen-Ju Zhu, Ya-Nan Li, Chao Xu, Jian-Ping Cao, Gui-Hua Zhao, Kun Yin

**Affiliations:** 1 National Institute of Parasitic Diseases, Chinese Center for Disease Control and Prevention (Chinese Center for Tropical Diseases Research); Shandong Institute of Parasitic Diseases, Jining, 272033 China; 2 Key Laboratory of Parasite and Vector Biology, National Health Commission of the People’s Republic of China, Shanghai, 200025 China; 3 School of Public Health, Shandong First Medical University & Shandong Academy of Medical Sciences, Jinan, 250117 China

**Keywords:** *Toxoplasma gondii*, Cerebral cysts, Differentially expressed transcripts, Nanopore RNA-seq, Mental and behavioral disorders

## Abstract

**Background:**

*Toxoplasma gondii* can cause severe damage to immunodeficient hosts, and also compromise brain structure and function in immunocompetent hosts during latent infection. In China, the two different isolates, *Chinese I* (*ToxoDB#9*) and *Chinese III* are dominant epidemic strains widely spreading in humans and domestic animals and can lead to latent infection in host brain tissues, but the comparison of their manipulation patterns and mechanisms remains unclear.

**Methods:**

Tachyzoites of the *TgWh6* (Wh6) strain and the *TgCtLHG* (LHG) strain were used for establishing in vitro infection models within mouse microglia BV2 cells, and the differences in their invasion and proliferation patterns were observed. C57BL/6 J mice were used to establish in vivo latent infection models. After behavioral tests, the differential expressed transcripts (DETs) of the infected and control animals’ cerebral cortex were sequenced by Nanopore RNA-seq. Functional differences of DETs were analyzed by Gene Ontology enrichment analysis (GO), Kyoto Encyclopedia of Genes and Genomes enrichment analysis (KEGG), and protein–protein interaction (PPI) and cluster analysis. Expression of the key candidates were verified by quantitative polymerase chain reaction (qPCR).

**Results:**

In our infection models, we found that Wh6 had more vigorous invasion and proliferation abilities in vitro, while LHG had a greater ability to form cysts in vivo. In the latent infection phase, behavioral changes, including spatial working memory, cognitive and motor abilities, and anxiety, were observed in both Wh6 and LHG infected mice; however, the LHG group showed more serious anxiety. Among DETs, genes related to major histocompatibility complex (MHC) class II molecules were significantly upregulated in the infected mice, while genes related to synaptic transmission and neurodegenerative diseases were downregulated in the infected groups. The downregulated DETs of *Sept4*, *Kcng4*, *Unc13c*, and *Prkcg* in the WH6 group, which are related to synaptic transmission, and *Ndrg2* and *Arc* in the LHG group, which are related to neurodegenerative diseases, were selected to be the key candidates in the latent infection phase.

**Conclusions:**

Compared with WH6, although LHG has a milder invasion ability, it can cause increased behavioral disorders in hosts. Genes related to synaptic transmission and neurodegenerative diseases may be the main causes of host mental and behavioral disorders.

**Graphical Abstract:**

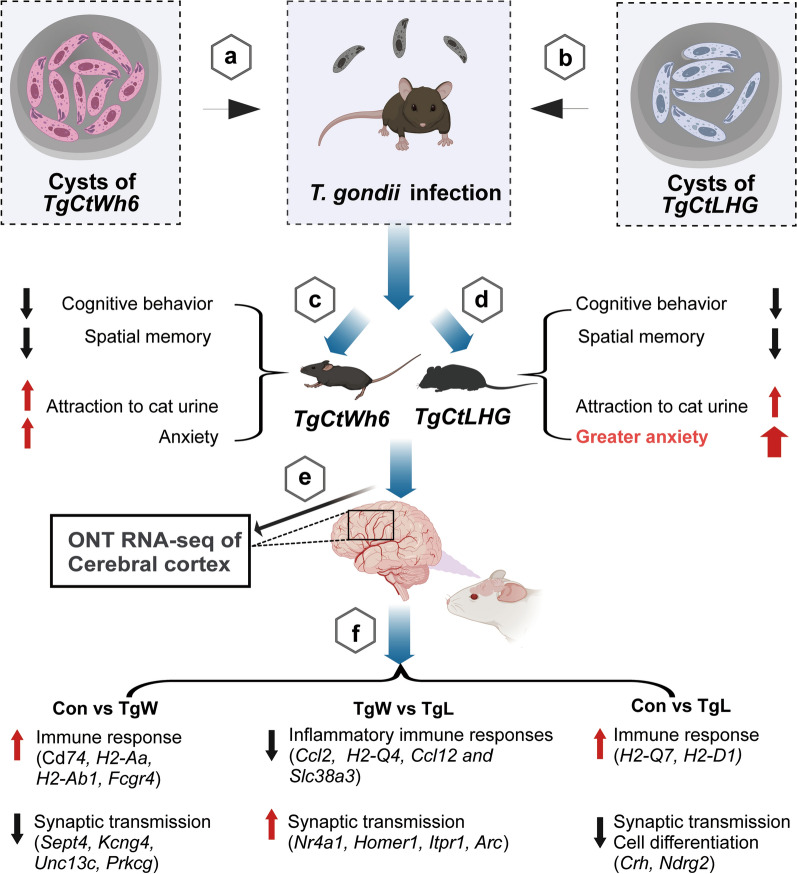

**Supplementary Information:**

The online version contains supplementary material available at 10.1186/s13071-025-06819-7.

## Background

*Toxoplasma gondii* is an intracellular apicomplexan parasite infecting nearly all warm-blooded animals worldwide [1, 2]. About 30–50% of the global population has been exposed to or infected with *T. gondii* [3]. *T. gondii* is highly prevalent in Western Europe, South America, and Africa [4, 5], with infection rates ranging from 10% in the USA to > 50% in France, Colombia, and Brazil [6–8]. Moreover, a 2018 survey on toxoplasmosis in China demonstrated that the *T. gondii* infection rate among the Chinese population was 8.2%, and 23.7% among livestock [9]. Because of its high prevalence and widespread distribution in its intermediate hosts, including livestock, poultry, and pets, *T. gondii* is increasingly recognized as a considerable risk to human health and the livestock industry.

Tachyzoites could rapidly invade host’s blood and various organs including eyes and internal organs, resulting in serious acute infections, particularly in immunodeficient patients. For instance, toxoplasmosis-induced encephalitis is a common cause of death in patients with acquired immunodeficiency syndrome (AIDS) [10]. Although the clinical symptoms in infected individuals with normal immune function are not apparent in the acute phase, tachyzoites could also transform into bradyzoites and tissue cysts in the latent phase. Notably, cerebral cysts, the most common *T. gondii*-associated tissue cyst type, could persistently infect brain tissues, leading to mental and behavioral disorders [11, 12]. In rodent infection models, it can trigger various behavioral disorders including loss of natural fear, an increased desire to explore, and a decline in cognitive function [13–15]. Further, it has been proved that neuroinflammation and synaptic alterations were associated with abnormal behavior post-chronic infections with *T. gondii* strains, for example, the synaptic proteins EAAT2 and GABAAα1, which are involved in excitation/inhibition balance in the central nervous system (CNS), could be downregulated after infection, and accompanied with increased microglia activation [16, 17]. In some cases, different phenotypes such as depression, anxiety, and reduced motor capacity have also been reported [18–20]. So far, there are already more than 300 publications that have shown that various types of psychiatric disorders, ranging from schizophrenia to depression, suicidal tendencies, Alzheimer’s disease, road rage-induced traffic accidents, and even positive behaviors (e.g., entrepreneurship and risk-taking) are significantly associated with *T. gondii* infection [21, 22]. However, the mental behavioral manipulation patterns caused by different *T. gondii* strains with different genotypes, as well as the different pathogenic characteristics, particularly for Chinese *T. gondii* isolates, still remain unclear [13, 14, 23].

To date, 12 genotypes of Chinese *T. gondii* strains have been identified, and the dominant strain, *Chinese I* (*ToxoDB#9*), accounts for 66.36% of all genotypes, followed by type I and II (variants) [24–26]. In particular, the *Chinese III* strain, also widespread in humans and domestic animals, is phylogenetically close to the typical type I virulent strains such as RH and GT1 [27]. Although its virulence is weaker, it is more likely to form brain cysts than *Chinese I* isolates [28, 29]. However, no study has reported the phenotype of behaviors of hosts infected with *Chinese III* strains nor has compared the manipulation patterns and mechanisms of different Chinese *T. gondii* isolates.

Therefore, in the present study, we have established both in vitro and in vivo infection models with *TgCtwh6* (Wh6; feline-sourced I × II hybrid strain) and *TgCtLHG* (LHG; human-sourced type III strain), respectively. On the basis of these, we have analyzed the differences in host symptoms in both acute and latent infection phases and assessed the behavioral differences as well as the full-length cerebral cortex transcript expression before and after chronic infection. Our findings may provide a basis for an in-depth interpretation of parasite-mediated mental and behavioral manipulation mechanisms with different genotypes.

## Methods

### Animals and parasites

*TgCtWh6* (Wh6, a Chinese I ×  II hybrid genotype strain, isolated from a stray cat in Wuhan, China) and *TgCtLHG* (LHG, a type III genotype strain, isolated from the peripheral blood of a patient with toxoplasmosis) were kindly provided by Prof. Jilong Shen at the Key Laboratory of Microbiology and Parasitology, Anhui Medical University (Hefei, Anhui, China). Brain cysts of Wh6 and LHG were continuously transmitted to the animal hosts BALB/c mice via cyst gavage [30].

Specific pathogen-free C57BL/6J mice, aged 6–8 weeks, were purchased from Pengyue Experimental Animal Breeding [Jinan, Shandong, China; animal license number: SCXK(Lu) 20220006]. All animals were maintained under a constant temperature (22 °C ± 2 °C) and humidity (55% ± 5%) under a 12 h day–night cycle, with free access to food and water. The drinking water was sterilized through high-pressure disinfection, and the feed and bedding were sterilized using ultraviolet light.

### Cell culture and in vitro infection

BV2 cells were routinely cultured in high-glucose Dulbecco’s modified Eagle medium (DMEM) containing 10% fetal bovine serum (FBS) (Gibco, USA) at 37 °C in a humidified atmosphere of 5% CO_2_. When the passage density was reached, all cells were digested with 0.25% EDTA–trypsin, divided into flasks at a 1:2 ratio, and cultured at 37 °C under 5% CO_2_ until 70–80% of the cells fused. Next, the medium was replaced with fresh high-glucose DMEM containing 3% fetal bovine serum. After 12 h, the cells were used in an in vitro experiment with *T. gondii* tachyzoites. In total, six-cell culture dishes (35 mm × 10 mm) were placed in sterile conditions and numbered, and 2.4 × 10^5^ cells were inoculated into each dish. After 12 h of culture at 37 °C under 5% CO_2_, the medium was replaced, and the cells were allowed to grow to 85% confluency. Tachyzoites were co-cultured with BV2 cells at a 1:2 ratio, and the infected cells were observed for invasion status at 12, 24, 48, and 72 h after infection.

### Construction of a chronic infection mouse model

In total, 60 female and 60 male C57BL/6J mice, aged 6–8 weeks, were used to establish chronic infection models. Animals were divided into three groups: control group (Con), Wh6-infected group (TgW), and LHG-infected group (TgL). Each group included 20 female and 20 male mice. Each infection group was gavaged with 0.2 mL of cerebral tissue suspension containing ten cysts, and the control group was gavaged with 0.2 mL of saline. After 45 days of inoculation, one mouse from each group was randomly selected and euthanized to obtain brain tissue. Cysts were observed under a microscope to determine infection effectiveness.

### Clinical observation

We observed clinical symptoms exhibited by tachyzoite-infected mice daily over 0–42 days after infection. For each infected mouse, the onset and duration of acute symptoms, such as back arching, erect hair, tremors, limb weakness, and eye disturbances, were recorded. The sex, survival time, and number of dead mice were also documented.

### Behavioral testing

#### Y-maze test (YM)

Each mouse was placed in the starting arm of a Y-maze and allowed to explore the three arms. The exploration behavior of a test mouse was recorded by a camera; in particular, we recorded the total distance traveled, the number of entries into each arm, and spontaneous alternation [= (total number of alternations/number of maximum alternations) × 100].

#### Open-field test (OFT)

An OFT was conducted using a camera to detect the movements of animals in a 50 × 50 cm^2^ arena in a quiet, soundproof, dimly lit (≈20 lx) room. A mouse was placed in the center of the arena and allowed to move freely for 10 min. The movements of each mouse in each of the three groups were recorded using an automatic video tracking system for 1 min. In particular, we recorded the total distance traveled, average speed, immobility time, number of entries into the central area, distance traveled within the central area, and time spent in the central area.

#### Predator odor-induced OFT

This test was used to evaluate the host’s fear response to predators. The method was similar to the OFT, but the urine of cat was placed sequentially in one corner of each compartment to assess how much time the mice spent in the urine-exposed areas.

### Brain tissue collection and *T. gondii* cyst counting

After 45 days of infection, the mice were anesthetized, euthanized, and immediately dissected to remove the whole brain. The brain tissue was washed with sterile phosphate-buffered saline (PBS) and placed in a Keeper preservation solution.

A random sample of mice from the infected groups was euthanized under sterile conditions with ether anesthesia, and the whole brain tissue was segmented along the longitudinal axis into left and right hemispheres, with the cerebral cortex, hippocampus, and olfactory bulb separated. Each part was thoroughly homogenized in 2 mL of PBS. Next, 10 µL of the homogenate was used to prepare a smear. The number of *T. gondii* cysts was counted under a microscope (400× magnification), and for each mouse, an average value was calculated from three smears.

### RNA extraction

After completion of all behavior tests, the whole brains of all tested mice were excised and immediately frozen in liquid nitrogen. Since cysts are predominantly localized in cortical areas of the brain [31], the whole cortical regions of a tested mouse were separated and used to be an RNA sample. The total RNA in each sample was extracted using TRIzol reagent (Thermo Fisher, USA), according to the manufacturer’s instructions. The total absorbance (A = A_260_/A_280_ + A_260_/A_230_) was detected to analyze the purity of RNA, and the Aglient 2100 Bioanalyzer was used to detect RNA concentration and integrity.

### Library construction and Nanopore RNA sequencing

We used Oxford Nanopore Technologies-based single-molecule real-time sequencing technology (i.e., ONT RNA-seq) for sequencing full-length RNA transcripts. Total RNA was extracted from mouse brain tissue, and mRNA was obtained through magnetic bead enrichment. The library was subsequently constructed, and the high-throughput sequencing was performed by Biomarker Technologies Co., Ltd. The raw sequence was evaluated for quality and filtered to remove adapter sequences, short segments, and low-quality sequences; finally, high-quality read lengths were analyzed, and data on differential representative transcripts were obtained.

### Functional enrichment analysis

Differentially expressed transcriptome data were obtained through quantitative analysis of transcriptome data from the brains of infected and control mice. The resulting sequences of differently expressed transcripts were compared with the Gene Ontology (GO) and Kyoto Encyclopedia of Genes and Genomes libraries (KEGG) to obtain relevant functional annotations and differentially expressed transcripts (DETs). To understand the changes in transcription levels in infected mice, we analyzed DETs with the following conditions: fold change (i.e., fold differences in DETs expression) is ≥ 1.5, and *P* (i.e., significance screening index for DETs) is < 0.05. The classification and enrichment of DETs were performed with GO functional annotation and KEGG pathway analyses in order to select the top ten upregulated and top ten downregulated DETs. The *P* values for these analyses were calculated using edge R, and *P* ≤ 0.05 was considered to indicate statistically significant enrichment.

### Protein–protein interaction and cluster analysis

The potential interactions between the encoded proteins were retrieved from the string database, the protein–protein interaction (PPI) networks were constructed, and the clustering analysis of protein populations was performed using clustering options [32].

### Statistics analysis

All data, expressed as means ± standard errors of the means (SEMs), were analyzed using GraphPad Prism (version 8.0). We used the Shapiro–Wilk normality test followed by Student’s *t*-test to compare the data from the two groups. Moreover, one-way analysis of variance (ANOVA) analysis followed by the post hoc Tukey test was used to analyze the effects of *T. gondii* infection. *P* < 0.05 was considered to indicate statistical significance.

## Results

### Wh6 tachyzoites are more invasive and proliferative than LHG tachyzoites within glial cells in vitro

**Fig. 1:**
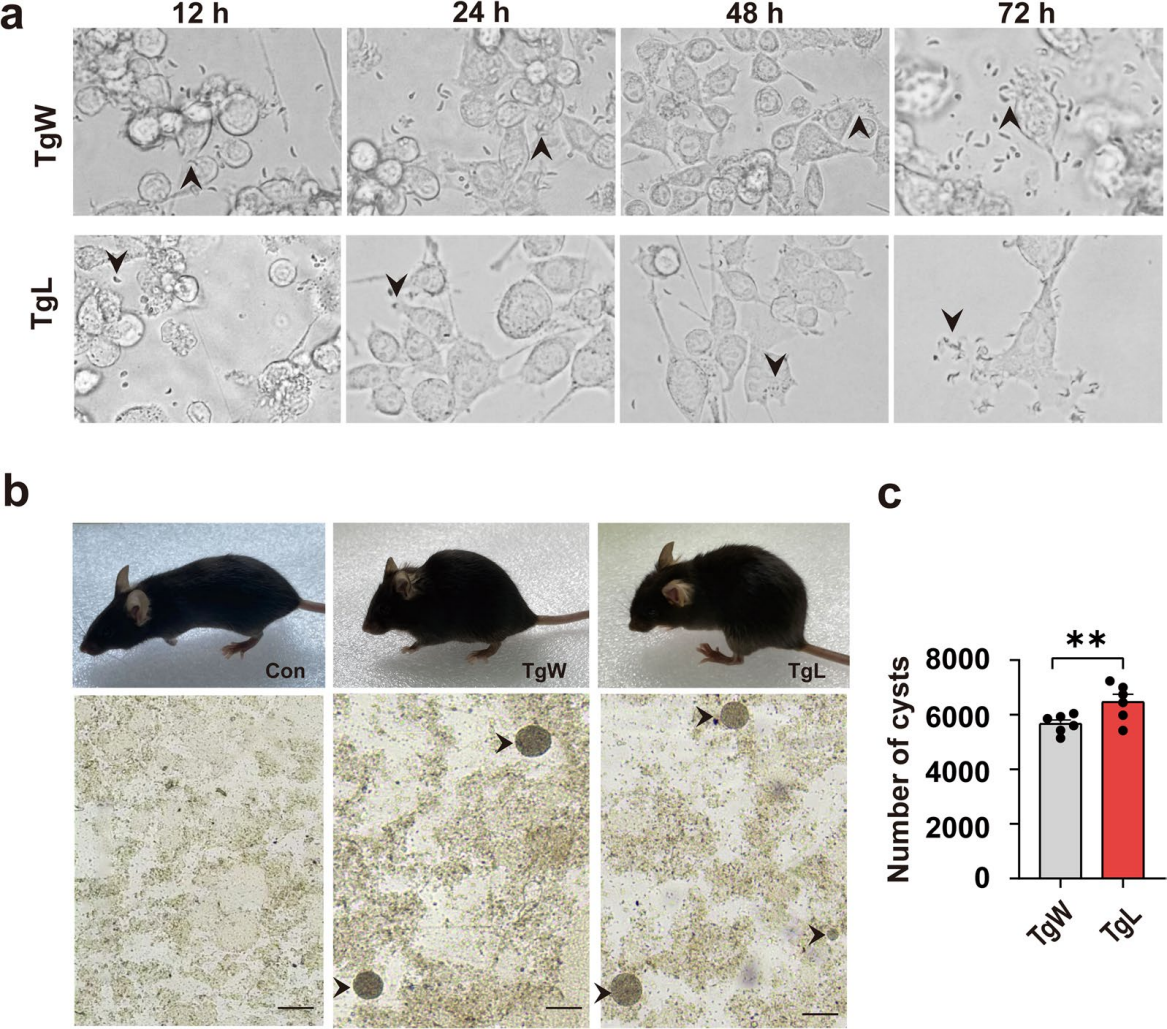
Invasion patterns of Wh6 and LHG tachyzoites in in vitro BV2 cell models and in vivo C57BL/6J mouse models. **a** Wh6 and LHG proliferation after co-cultured with BV2 cells for 12, 24, 48, and 72 h. Arrows indicate typical *T. gondii* tachyzoites invading cells during the co-culturing period. **b** Infected mice and cysts formed in mouse brain tissues (magnification: 100×; scale bar: 50 μm). Arrows indicate typical cysts in the brain tissue. **c** Comparison of the number of cysts between Wh6 and LHG infected brain tissues. Values are presented as means ± SEMs. **P < 0.01

By continuous detection under an inverted microscope, most tachyzoites of either strain could adhere to BV2 cell surfaces at 12 h of co-culturing, with only a few of them entering the cytoplasm. Almost all of the tachyzoites could complete cell invasion at 24 h after infection. At 48 h of co-culturing, the parasites began to divide, some Wh6-infected BV2 cells demonstrated a protrusion filled with the parasites on their membranes. By 72 h after infection, many parasites were released and began to start a new round of invasion (Fig. 1a, upward arrow). However, the tachyzoites of LHG could not lead to cell membrane protrusion or parasite release until 72 h after co-culturing (Fig. 1a, downward arrow). Thus, our results showed that Wh6 has stronger invasive and proliferative ability in BV2 cells than LHG.

### The infection symptoms caused by Wh6 and LHG tachyzoites, and the number of brain cysts were different

At 5 days after infection, the infected mice began to exhibit acute symptoms such as hunched back, piloerection, tremor, limb weakness, eye discoloration, and decreased activity (Fig. 1b). This acute onset phase lasted for 20 days after infection. In general, TgW mice tended to have more severe eye damage, while TgL mice were more likely to demonstrate hind limb weakness (Table 1). We found that all infected mice demonstrated cyst formation in brain tissues at 21 days after infection (Fig. 1b), but the number of brain cysts in the TgL group was significantly more than those in the TgW group (Fig. 1c). Surprisingly, we also found that all of the dead mice were female, and most of them belonged to the TgL group (Table 1). As a result, we speculated that LHG strain may have a sex bias for host fatality.

**Table 1:** Continuous symptom observations of mice in the TgW and TgL groups (*n* = 20)

Mouse sex	*T. gondii* strain	Survival (days)	Death rate^a^ (%)	Acute symptom rate (%)				
				Hogback	Piloerection	Shiver	Hind leg weakening	Whitening of eyes
Male	Wh6	–	0 (0/20)	100 (20/20)	50 (11/20)	100 (20/20)	0 (0/20)	12.5 (2/20)
	LHG	–	0 (0/20)	100 (20/20)	50 (12/20)	100 (20/20)	100 (20/20)	0 (0/20)
Female	Wh6	16	10.0 (2/20)	100 (20/20)	87.5 (18/20)	100 (20/20)	0 (0/20)	0 (1/20)
	LHG	12	65.0 (13/20)	100 (20/20)	100 (20/20)	100 (20/20)	100 (20/20)	0 (0/20)

^a^Death rate = (number of deaths/number of infected individuals × 100%)

### Different genotypes of *T. gondii* strains cause different types of mental and behavioral disorders

#### The Y-maze results showed that chronic *T. gondii* infection impaired the spatial working memory ability of mice

**Fig. 2:**
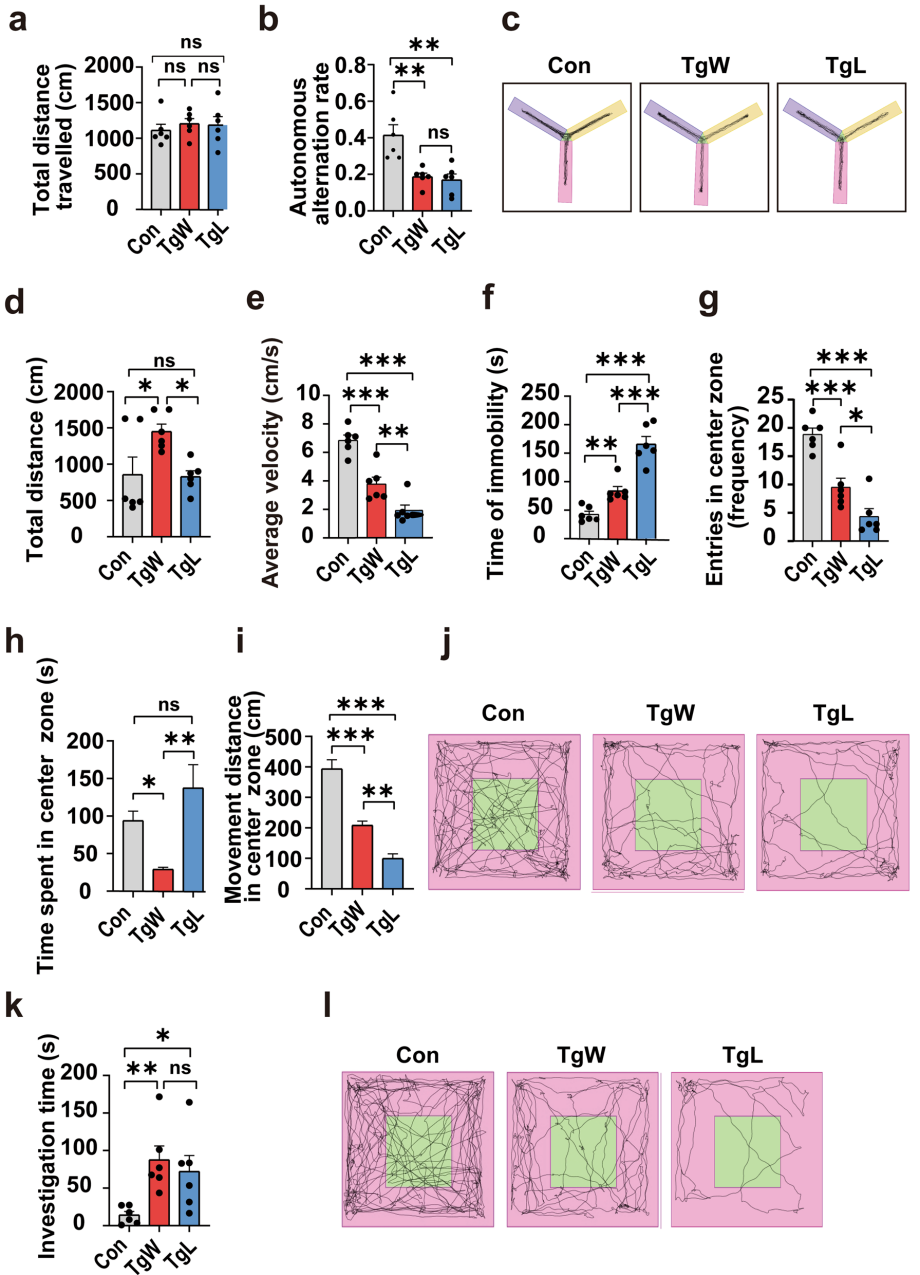
The behavior analysis results of mice infected with or without the WH6 and LHG strain of *T. gondii*. Total distance traveled (**a**, *F* = 0.274, *P* = 0.764, LSD test), autonomous alteration rate (**b**, *F* = 11.757, *P* = 0.001, LSD test), and motion trajectory diagram (**c**). Results of OFT without provocation. Total distance (**d**), average velocity (**e**), time of immobility (**f**), frequency of entries into the central zone (**g**), distance moved in the central zone (**h**), time spent in the central zone (**i**), and motion trajectory diagram (**j**). Investigation time of OFT with cat urine. Investigation time (**k**), and motion trajectory diagram (**l**). Data are presented as means ± SEM. ***P < 0.001, **P < 0.01, *P < 0.05

Although the total distance traveled in the YM test did not differ significantly among the TgW, TgL, and Con groups (Fig. 2a), the autonomous alternation rate decreased significantly in both infected groups compared with the Con group (*P* < 0.01; Fig. 2b), indicating that both WH6 and LHG could impair the spatial working and short-term memory ability of the hosts in the chronic infection phase, with a similar degree. A representative motion trajectory diagram is shown in Fig. 2c.

#### The OFT results showed that the TgL group had more severe motor impairments and higher anxiety levels

Compared with the Con group, both TgW and TgL groups exhibited significantly lower average speeds and longer immobility times; however, the TgW group showed significantly longer total distance traveled (*P* < 0.05 or *P* < 0.001; Fig. 2d–f), and an increase in the total number of entries into the central zone but a reduction in the total distance traveled and time spent in the central zone (*P* < 0.01 or *P* < 0.001; Fig. 2g–i). Thus, the chronic infection may cause slight motor impairments along with anxiety-like behavior in the hosts. Compared with TgW, the TgL group demonstrated a slightly shorter total distance (*P* < 0.05), lower average speed (*P* < 0.01), and significantly higher immobility time (*P* < 0.001; Fig. 2d–f), indicating that LHG strain had relatively more severe motor impairments, consistent with our results in the acute infection phase. In particular, mice in the TgL group showed fewer central zone entries but slightly longer time spent in the central zone (*P* < 0.05 or *P* < 0.01; Fig. 2g–i), suggesting that LHG strain may be more likely to cause hosts’ anxiety. A representative motion trajectory diagram is shown in Fig. 2j.

#### The cat urine OFT results showed that both TgW and TgL mice had the loss of natural fear of predators, while the TgW group was more significant

We collected urine samples from domestic cats and conducted a predator-induced mineshaft OFT. The results showed that all the infected mice spent significantly longer time in the urine-containing area than the control mice (Fig. 2k), consistent with the previous results [31, 33]. However, mice in the TgW group appeared to have a more significant degree of fear loss (*P* < 0.01) than those of the TgL group (*P* < 0.05). A representative motion trajectory diagram is shown in Fig. 2l.

### Identification of DETs among TgW, TgL, and Con groups

**Fig. 3:**
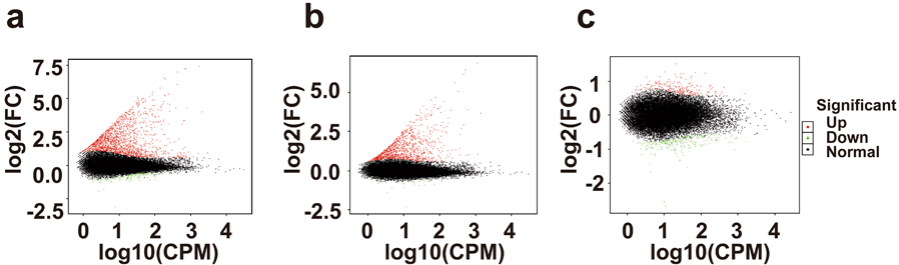
MA plots of DETs in Con versus TgW (**a**), Con versus TgL (**b**), and TgW versus TgL (**c**) groups

The third-generation high-throughput ONT RNA-seq results demonstrated that the TgW and TgL groups included 1789 and 1119 DETs compared with the Con group, respectively. The TgW and TgL groups included 1659 and 1089 upregulated DETs, respectively; and 130 and 30 downregulated DETs, respectively. In the comparison of the TgW and TgL groups, 466 DETs were detected, of which 223 were upregulated and 243 were downregulated, respectively (Fig. 3).

### Chronic infection can intensively influence the immune response and synaptic transmission-related processes of the host

**Fig. 4:**
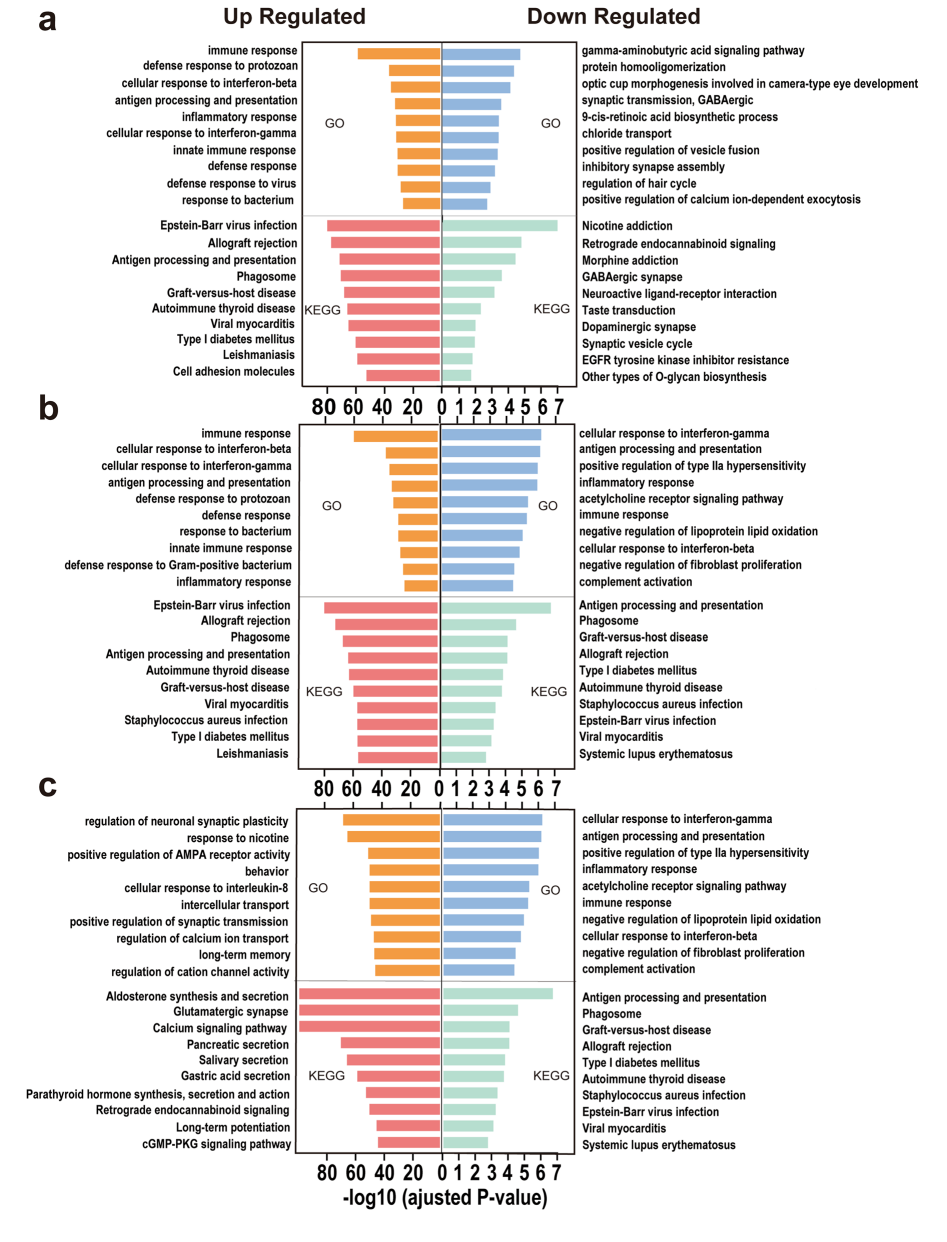
Top ten upregulated and downregulated DETs as shown by GO and KEGG enrichment analysis in Con versus TgW (**a**), Con versus TgL (**b**), and TgW versus TgL (**c**) groups

Compared with the Con group, the top ten upregulated biological processes (BPs) in the TgW group were immune response, response to protists, cellular response to interferon β, antigen processing and presentation, inflammatory response, cellular response to interferon γ, innate immune response, defense response, defense response to viruses and response to bacteria. The top ten upregulated KEGG pathways were Epstein–Barr (EB) virus infection, allogenic transplant rejection, antigen processing and presentation, phagosome, xenograft host defense, autoimmune thyroid disease, viral myocarditis, type I diabetes, leishmaniasis and cell adhesion molecules (Fig. 4a, left). The top ten downregulated BPs were the gamma-aminobutyric acid (GABA) signaling pathway, protein homo-oligomerization, morphogenesis of camera-shaped eye development, GABAergic synaptic transmission, 9-cis retinoic acid biosynthesis, chloride ion transport, vesicle fusion-positive regulation, inhibitory synapse assembly, hair cycle regulation and calcium-dependent exocytosis positive regulation. The top 10 downregulated KEGG pathways were nicotine addiction, retrograde endogenous cannabinoid signaling, morphine addiction, GABAergic synaptic transmission, neuroactive ligand–receptor interactions, taste transduction, dopaminergic synapses, synaptic vesicle cycles, epidermal growth factor receptor tyrosine kinase inhibitor resistance and other types of o-glycan biosynthesis (Fig. 4a, right). Taken together, these results demonstrated that infection with Wh6 mainly leads to upregulation of processes involved in immune responses associated with pathogenic infection and downregulation of processes related to synaptic signaling.

Simultaneously, compared with the Con group, the top ten upregulated BPs in the TgL group were immune response, cellular response to interferon β, cellular response to interferon γ, antigen processing and presentation, response to protists, defense response, response to bacteria, innate immune response, defense response to gram-positive bacteria, and inflammatory response. The top ten upregulated KEGG pathways were EB virus infection, allogenic transplant rejection, phagosome, antigen processing and presentation, xenograft host defense, viral myocarditis, *Staphylococcus aureus* infection, type I diabetes, and leishmaniasis (Fig. 4b, left). The top ten downregulated BPs were the regulation of platelet-derived growth factor generation, regulation of vascular endothelial growth factor generation, negative regulation of the extracellular signal-regulated kinase 1 and 2 cascades, negative regulation of cell growth, membrane invagination, synaptic vesicle cycle, vesicle-mediated transport in synapses, positive regulation of adipocyte differentiation, negative regulation of hepatocyte differentiation, and negative regulation of forebrain neuron differentiation. The top ten downregulated KEGG pathways were alcohol addiction, chemokine signaling pathways, human cytomegalovirus infection, HIV type I infection, GABAergic synapses, circadian rhythms, opioid addiction, serotonergic synapses, cholinergic synapses, and glutamatergic synapses (Fig. 4b, right). Similarly, these results indicated that infection with LHG would also lead to the upregulation of immune responses, and the downregulation of synaptic signaling, as well as processes related to cell differentiation.

### LHG strain may cause enhanced variation of synaptic transmission and metabolic secretion

Compared with the TgW group, the top ten upregulated BPs in the TgL group were the regulation of neuronal synaptic plasticity, response to nicotine, positive regulation of α-amino-3-hydroxy-5-methyl-4-isoxazole-propionic acid (AMPA) receptor activity, behavior, intracellular transport, cellular response to interleukin 8, positive regulation of synaptic transmission, regulation of calcium ion transport, long-term memory, and regulation of cation channel activity. The top ten upregulated KEGG pathways were aldosterone synthesis and secretion, glutamatergic synapses, calcium signaling pathways, pancreatic secretion, salivary secretion, gastric secretion, cosecretion and action of parathyroid hormones, retrograde endocannabinoid signaling, long-term potentiation, and cyclic guanosine monophosphate-protein kinase G (cGMP-PKG) signaling pathways (Fig. 4c, left). The top ten downregulated BPs were the cellular response to gamma interferon, antigen processing, and presentation, positive regulation of type II hypersensitivity reactions, inflammatory responses, acetylcholine receptor signaling pathways, immune responses, lipoprotein lipid oxidation negative regulation, cellular response to interferon β, fibroblast proliferation negative regulation, and complement activation. The top ten downregulated KEGG pathways were phagosomes, plant–pathogen interactions, allograft rejection, type 1 diabetes, autoimmune thyroid disease, *S. aureus* infection, EB virus infection–viral myocarditis, and systemic lupus erythematosus (Fig. 4c, right). These results demonstrated that the regulation of hosts’ synaptic transmission and metabolic secretory function may be enhanced by the LHG strain, yet the inflammatory immune responses showed more weakness in the TgL group.

### Verification of the key DET candidates

**Fig. 5:**
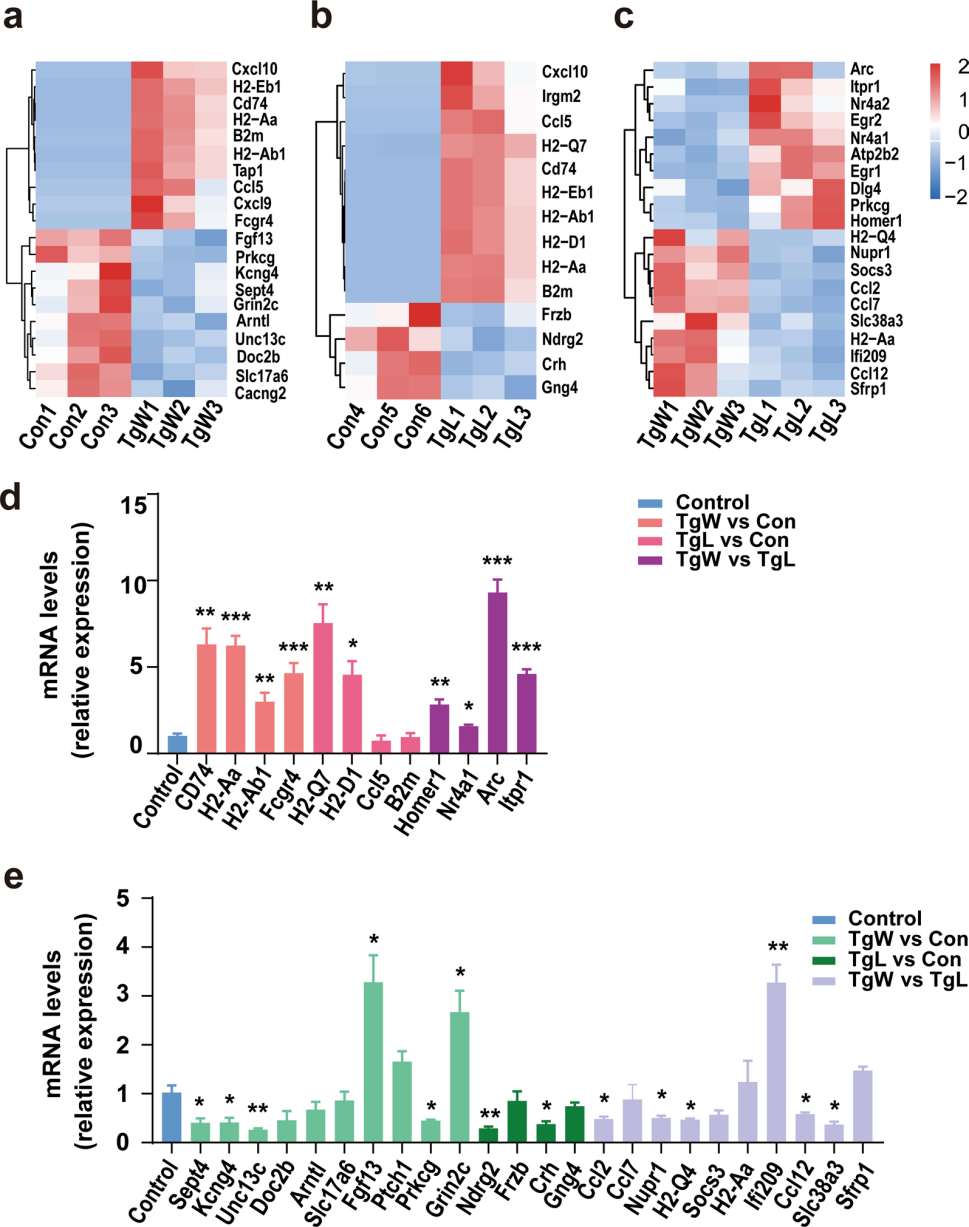
Expression heatmaps of genes corresponding to key DETs and qPCR validation results of key candidates genes. Gene expression profiles of upregulated and downregulated DETs in Con versus TgW (**a**), Con versus TgL (**b**), and TgW versus TgL (**c**) groups. qPCR results of the upregulated (**d**) and downregulated key candidate DETs (**e**). Data are presented as means ± SEMs. ***P < 0.001, **P < 0.01, *P < 0.05

DETs with high expression changes, top GO annotation and top KEGG enrichment were selected to obtain heatmaps of gene expression profiles corresponding to the top ten key candidate DETs (Fig. 5a–c). Among them, the first four upregulated key candidates were selected for qPCR verification in each group, and the results showed that most of the gene expression was consistent with our ONT RNA-seq results (Fig. 5d and Additional file 1: Table S1). In particular, the mRNA expression of *H2-Aa* and *Fcgr4* in the TgL group, *H2-Q7* in the TgL group, and *Arc* and *Itpr1* in the TgW versus TgL group were significantly upregulated.

Similarly, the verification of downregulated key candidates showed that a majority of the selected gene expression results were consistent with the DETs results (Fig. 5e and Additional file 2: Table S2). The expression of *Sept4*, *Kcng4*, *Unc13c*, and *Prkcg* genes in the TgW group; *Edrg2* and *Crh* genes in the TgL group; and *Ccl2*, *Nupr*, *H2-Q4*, *Ccl12*, and *Slc38a3* genes in the TgW versus TgL group were significantly downregulated.

### Wh6 and LHG strains may manipulate host behaviors with different patterns of gene regulation

**Fig. 6:**
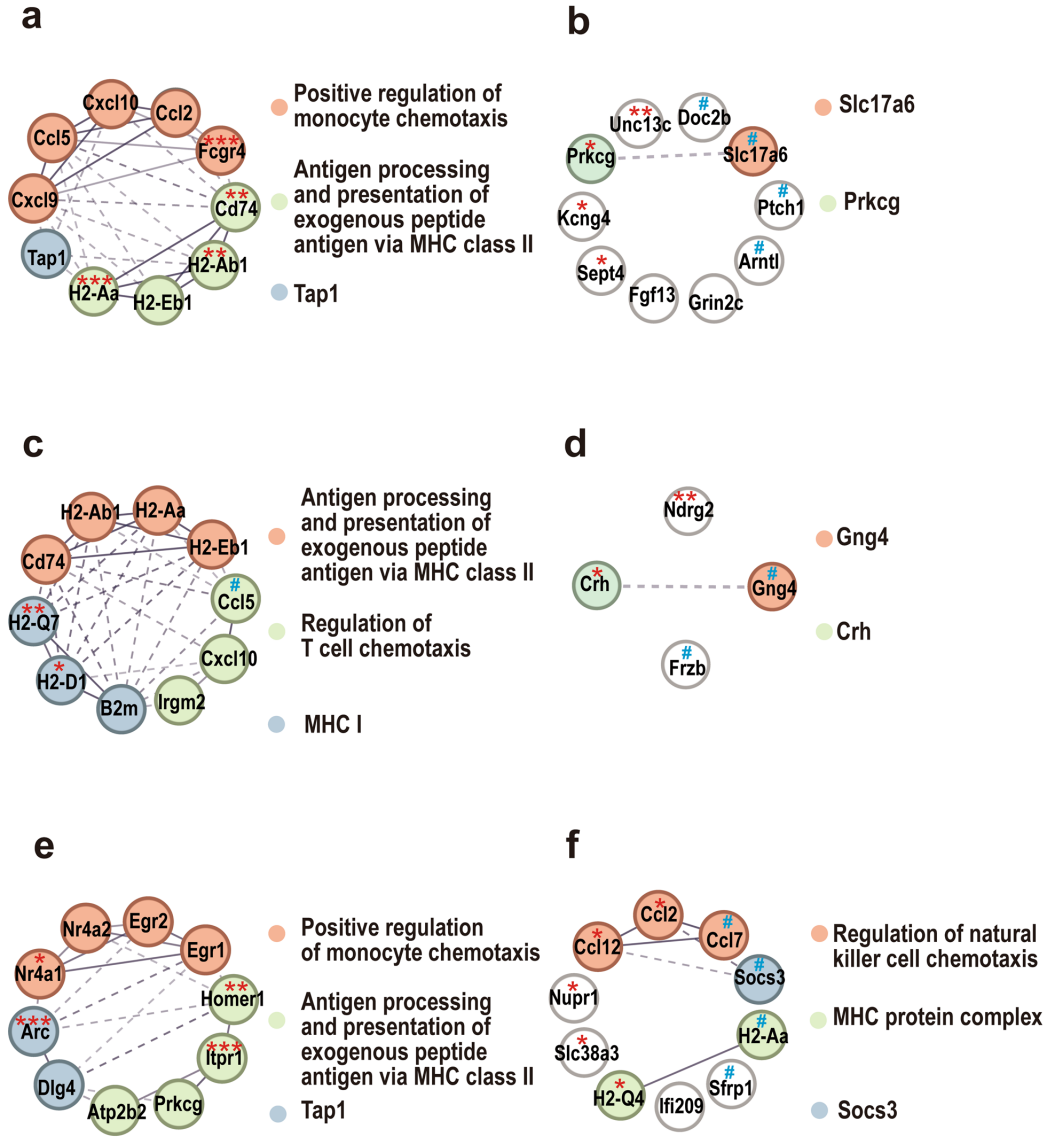
PPI network of DET-encoded proteins. Upregulated DETs in Con versus TgW (**a**), Con versus TgL (**c**), and TgW versus TgL (**e**) groups. Downregulated DETs in Con versus TgW (**b**), Con versus TgL (**d**), and TgW versus TgL (**f**) groups. * Represents genes of key candidate DETs validated by qPCR; # denotes genes with no changes in key candidate DETs validated by qPCR

On the basis of our PPI results, we found that compared with the Con group, the upregulated key candidates *Cxcl10*, *Cxcl0*, *Ccl5*, *Ccl2*, and *Fcgr4* in the TgW group were related to the positive regulation of monocyte chemotaxis, the *H2-Eb1*, *H2-Aa*, *H2-Ab1*, and *Cd74* were related to MHC class II-mediated exogenous antigen processing and presentation, and *Tap1* was related to antigen processing and transport (Fig. 6a). The downregulated key candidates *Unc13c*, *Prkcg*, *Sept4*, *Kcng4*, etc., were all related to the function of synaptic transmission (Fig. 6b).

Whereas in the TgL group, compared with the Con group, although the upregulated key candidates *H2-Aa*, *H2-Eb1*, *H2-Ab1*, and *Cd74* were related to MHC class II-mediated exogenous antigen processing and presentation; *H2-Q7*, *H2-D1*, and *B2m2* were related to MHC class I molecules. Furthermore, *Ccl5*, *Cxcl0*, and *Irgm2* were related to the regulation of T-cell chemotaxis (Fig. 6c). Moreover, the downregulated key candidates, such as *Crh* and *Ndrg2*, were related to neurodegenerative diseases (Fig. 6d).

Particularly, compared with the TgW group, the upregulated key candidates *Nr4a1*, *Nr4a2*, *Egr1*, and *Egr2* in the TgL group were related to the cell response to corticotropin-releasing hormone stimulus, early growth response, *N*-terminal and NAB family, and nuclear glucocorticoid receptor binding. Simultaneously, *Homer1*, *Itpr1*, *Prkcg*, and *Atp2b2* were related to postsynaptic cytosol and platelet calcium homeostasis, and *Arc* and *Dlg4* were related to the regulation of synaptic plasticity (Fig. 6e). Further, the downregulated key candidates, such as *Ccl2*, *Ccl12*, and *Ccl7*, were related to the regulation of natural killer cell chemotaxis, and *Nupr1*, *Slc38a3*, *Ifi209*, and*Sfrp1* were related to the inhibition of SOCS3-mediated cell signaling and participate in the inhibition of inflammation (Fig. 6f).

## Discussion

*Toxoplasma gondii*, a typical representative manipulator parasite [34, 35], can significantly affect the psychological behavior of the host and thus threaten public health [36–38]. It has been proved that neurogliocytes could be continuously activated by *T. gondii* infection, and the infected mice could present a marked decrease in microglia population compared with the non-infected group, both with the ME49 clonal strain and the *TgCkBrRN2* atypical strain (CK2) [39]. Therefore, we have established in vitro infection models of Wh6 and LHG strains by using the BV2 cell line, to better understand their ability to invade microglia. Our current results demonstrate that Wh6 has a significantly more intensive ability than LHG in terms of microglia invasion and intracellular proliferation in vitro. Consistently, it has been proved that in the mice infected with the *TgCtWH6* strain, hosts developed cognitive behavioral disorders, neuronal apoptosis, and Aβ deposition; therefore, neuroinflammatory responses involved in microglia polarization may be the molecular and cellular mechanisms involved [40]. We have also found that the Wh6-infected mice showed significant cognitive impairment, speculating that the increased cognitive impairment caused by WH6 may be related to its stronger ability to invade microglia. Moreover, our results also found that LHG had a stronger brain cyst formation ability in the infected mice, as well as an increased anxiety-like behavioral disorder during the chronic infection phase. Thus, our results further remind us that there may be a direct correlation between the brain cyst formation ability and the degree of behavioral abnormalities.

Simultaneously, our ONT RNA-seq results revealed that the significantly upregulated DETs in the two infected groups were primarily related to immune response and pathogen infection defense-related pathways, also consistent with previous studies [41]. However, these studies mainly focused on the intensive virulent type I strain (e.g., RH) and the moderately virulent type II strains (e.g., Prugniaud and ME49) [42–44]. We believe our results would provide a necessary supplement for better understanding the mechanism of hosts’ mental and behavioral disorders manipulated by *T. gondii* strains prevalent in China with different genotypes.

Importantly, we also found that *T. gondii* strains with different genotypes could regulate host neuroinflammatory responses through different pathways. For instance, Wh6 and LHG may mediate the host neuroinflammatory response by upregulating different MHC class molecules. Further, our results showed that genes related to synaptic transmission function were primarily downregulated by Wh6, whereas genes related to neurodegenerative diseases were significantly downregulated by LHG. In addition, we found that the mortality of LHG-infected mice had a significant gender preference in the acute infection stage. As a matter of fact, it has been indicated that the mortality of female mice infected with *T. gondii* was generally higher than that of male mice, and the reason may be that female mice produce more inflammatory cytokines in response to *T. gondii* infection [45]. Our results precisely indicated that in the TgW versus TgL comparison group, the enrichment of response to IL-8 in the top ten GO analysis in the TgL group was more significant, indicating that the above hypothesis is reasonable.

As to the selected key candidates in the present study, the significant DETs in the different infected groups could also reflect the differences in the manipulation mechanism. For example, genes associated with positive regulation of monocyte chemotaxis, such as *Fcgr4*, *H2-Aa*, *H2-Ab1*, and *Cd74*, were upregulated in the TgW group, while genes associated with positive regulation of T-cell chemotaxis, such as *H2-Q7* and *H2-D1*, were upregulated in the TgL group. Since low-affinity immunoglobulin gamma Fc region receptor IV, *Fcgr4*, has been reported to be upregulated in leishmaniasis-infected mice, and ligation of FCGR4 by antigen-IgE(b) immune complexes could promote macrophage-mediated phagocytosis, antigen presentation to T cells, and proinflammatory cytokine production [46], it has also been used as a potential diagnostic marker for cutaneous leishmaniasis [47]. Secondly, the upregulated H-2 class I histocompatibility antigen, Q7 alpha chain, *H2-Q7*, belongs to the adaptive immune MHC I family response class, and it has been proved that it participates in foreign antigen presentation, endogenous processing, and presentation of process polypeptide antigens, and thus could promote the production of immune factors by T cells, enhancing adaptive immunity [48]. These key candidates indicate that the Wh6 strain was more likely to stimulate the phagocytosis and killing of the host’s central immune cells, whereas the LHG strain was more immunogenic to the host’s central immune system. This may be responsible for comprehending why LHG has a higher ability for brain cyst formation. Besides, there are several previous studies showed that the WH6 strain could cause neuroinflammation, synaptic loss, and cognitive deficits in C57BL/6J mice, and the behavioral disorder phenotype were perfectly consistent with our study [49, 50]. Simultaneously, in Tao et al.’s study, the tachyzoites of WH6 have been verified that they could indirectly induce APP protein production in HT22 by polarized BV2 cells, and the expression of CD80, pro-inflammatory factors, notch, and hes1 could be enhanced in the infected BV2 cells [49]. Moreover, the RNA-seq results in Wu et al.’s study [51] have also indicated that WH6 infection could trigger extensive neuroinflammation in the prefrontal cortex, and the related genes such as *Fcgr4*, *Ccl22*, and *H2-D1* were highly consistent with our present research.

As to the downregulated key candidates, genes related to synaptic transmission, such as *Unc13a*, *Prkcg*, *Sept4*, and *Kcng4* have been selected from the TgW group, while genes related to neurodegeneration, such as *Crh* and *Ndrg2* were selected from the TgL group. Firstly, it is well known that the protein unc-13 homolog C, *Unc13*, family is critical in regulating neurotransmitter release and synaptic plasticity in the CNS. *Unc13a* is involved in several related processes including glutamatergic synaptic transmission, synaptic vesicle maturation, and the regulation of synaptic transmission at parallel fiber–Purkinje cell synapses [52]. Secondly, *Ndrg2* is an *N*-myc downstream regulatory gene family member. It is primarily expressed in astrocytes and involved in the pathogenesis of many neurological diseases, such as stroke, neurodegenerative diseases (Alzheimer’s and Parkinson’s diseases), and psychiatric disorders (depression and attention deficit hyperactivity disorder) [53]. This may help to explain why the infected animals showed a loss of natural fear of predators, anxiety behaviors, and memory impairments.

Importantly, the expression of genes related to synaptic plasticity and neurological diseases such as *Arc*, *Homer1*, and *Itpr1* in the TgL group were significantly upregulated compared with the TgW group. Arc is an activity-regulated cytoskeleton-associated protein. It can regulate synaptic strength through multiple mechanisms and is essential for memory consolidation and learning [54]. Meanwhile, the expression of natural killer cell chemotaxis-related genes such as *Ccl2*, *Ccl12*, *H2-Q4*, and *Slc38a3* in the TgL group was lower in expression than in that of the TgW group. Particularly, CCL2, a ligand for C–C chemokine receptor CCR2, could act on dopaminergic neurons to increase their excitability, dopamine release, and motor activity, as well as increase NMDA-mediated synaptic transmission in neurons containing dopamine D1 and D2 receptors [55]. As a result, our finding may provide a further understanding of why LHG could induce more severe anxiety-like behaviors.

Mental and behavioral manipulation mechanisms of different *T. gondii* strains in their host’s are complex. Although our results promote a hypothesis that differences in neuroinflammatory responses and synaptic transmission may be the key pathways for the differences in the invasion and mental manipulation ability between the two *T. gondii* strains. However, accurate molecular mechanisms need to be further verified.

## Conclusions

Both the *Chinese I* Wh6 strain and the *Chinese III* LHG strain could cause cognitive and spatial memory impairment, as well as anxiety-like behaviors in the hosts, but the anxiety caused by LHG appeared more obvious. Immune response and synaptic transmission were significantly enriched and may be the major influenced pathways. Further, our results indicated that genes related to synaptic transmission such as *Unc13c*, *Prkcg*, *Sept4*, and *Kcng4* may be the main factors responsible for Wh6 mental and behavioral manipulation; while genes related to neurodegeneration, such as *Ndrg2* and *Arc*, as well as *Ccl2*, may be the key factors for explaining the stronger mental manipulation ability of the LHG strain (Graphical Abstract).

## Supplementary Information

Additional file 1: Table S1. Candidate key DETs corresponding to up-regulation genes in the brain tissue of Wh6 strain and LHG strain before and after infection.

Additional file 2: Table S2. Candidate key DETs corresponding to down-regulated genes in the brain tissue of Wh6 strain and LHG strain before and after infection.

## Data Availability

The data presented in this study are included in the article and its Supplementary Material. Further any further inquiries, please contact the corresponding author.

## Abbreviations

*T. gondii*: *Toxoplasma gondii*
Wh6: Chinese cat-derived type I *TgCtWh6* strain of *T. gondii*
LHG: Human type III *TgCtLHG* strain of *T. gondii*
DETs: Differentially expressed transcripts
GO: Gene Ontology
KEGG: Kyoto Encyclopedia of Genes and Genomes
PPI: Protein–protein interaction
qPCR: Quantitative polymerase chain reaction
AIDS: Acquired immunodeficiency syndrome
CNS: Central nervous system
DMEM: Dulbecco’s modified Eagle medium
FBS: Fetal bovine serum
Con: Control group
TgW: Wh6-infected
TgL: LHG-infected
OFT: Open-field test
YM: Y-maze
PBS: Sterile phosphate-buffered saline
SEMs: Standard errors of the means
ONT: Oxford Nanopore Technologies
BPs: Biological processes
EB: Epstein–Barr
GABA: Gamma-aminobutyric acid
HIV: Human immunodeficiency virus
AMPA: α-Amino-3-hydroxy-5-methyl-4-isoxazole-propionic acid
cGMP-PKG: Cyclic guanosine monophosphate-protein kinase G
MHC: Major histocompatibility complex
TAP1: Transporter associated with antigen processing 1
