# Supplementary material for: Effects of latent infection of Toxoplasma gondii strains with different genotypes on mouse behavior and brain transcripts
Source: Parasit Vectors. 2025 May 26;18:190. doi: 10.1186/s13071-025-06819-7 (PMC12107737; doi:10.1186/s13071-025-06819-7)
Supplement: Supplementary file 1 — Additional file 1: Table S1. Candidate key DETs corresponding to up-regulation genes in the brain tissue of Wh6 strain and LHG strain before and after infection. [file 13071_2025_6819_MOESM1_ESM.docx]

**Additional: Table S1** Candidate key DETs corresponding to up-regulation genes in the brain tissues of Wh6 strain and LHG strain before and after infection.

|  | **Gene Symbol** | **Full name of gene** | **Log_2_(FC)** | **P value** |
| --- | --- | --- | --- | --- |
| WH6 | *Cd74*** | CD74 antigen | 7.44 | 4.26E-263 |
|  | *H2-Aa**** | H-2 class II histocompatibility antigen, A-B alpha chain | 7.25 | 9.96 E-202 |
|  | *H2-Ab1*** | histocompatibility 2, class II antigen A, beta 1 | 7.22 | 3.52E-196 |
|  | *H2-Eb1* | histocompatibility 2, class II antigen E beta | 6.99 | 2.22E-172 |
|  | *Cxcl10* | C-X-C motif chemokine ligand 10 | 6.54 | 2.61E-101 |
|  | *Cxcl9* | C-X-C motif chemokine ligand 9 | 6.46 | 4.32E-77 |
|  | *Ccl5* | C-C motif chemokine ligand 5 | 5.80 | 1.20E-63 |
|  | *Tap1* | transporter 1, ATP-binding cassette, sub-family B | 5.15 | 3.37E-63 |
|  | *Ccl2* | C-C motif chemokine ligand 2 | 5.10 | 1.31E-45 |
|  | *Fcgr4**** | low affinity immunoglobulin gamma Fc region receptor IV | 5.00 | 2.08E-43 |
| LHG | *Cd74* | CD74 antigen | 6.87 | 4.44E-270 |
|  | *H2-Aa* | histocompatibility 2, class II antigen A, alpha | 6.44 | 1.31 E-206 |
|  | *H2-Ab1* | histocompatibility 2, class II antigen A, beta 1 | 6.13 | 1.12E-191 |
|  | *H2-Eb1* | histocompatibility 2, class II antigen E beta | 5.06 | 1.04E-82 |
|  | *Ccl5*^#^ | C-C motif chemokine ligand 5 | 4.67 | 3.71E-69 |
|  | *Cxcl10* | C-X-C motif chemokine ligand 10 | 4.19 | 7.14E-52 |
|  | *H2-Q7*** | H-2 class I histocompatibility antigen, Q7 alpha chain | 4.11 | 1.35E-52 |
|  | *H2-D1** | histocompatibility 2, D region locus 1 | 3.88 | 1.17E-112 |
|  | *B2m*^#^ | beta-2 microglobulin | 3.64 | 4.02E-111 |
|  | *Irgm2* | immunity-related GTPase family M member 2 | 3.81 | 4.06E-48 |
| WH6 vs LHG | *Homer1*** | homer scaffolding protein 1 | 1.12 | 2.05E-04 |
|  | *Arc**** | activity regulated cytoskeletal-associated protein | 1.09 | 2.36E-05 |
|  | *Nr4a1** | nuclear receptor subfamily 4, group A, member 1 | 1.25 | 1.93E-08 |
|  | *Nr4a2* | nuclear receptor subfamily 4, group A, member 2 | 1.13 | 1.74E-04 |
|  | *Egr1* | early growth response 1 | 1.23 | 1.23E-12 |
|  | *Atp2b2* | ATPase, Ca++ transporting, plasma membrane 2 | 1.21 | 3.00E-07 |
|  | *Egr2* | early growth response 2 | 1.13 | 1.06E-04 |
|  | *Itpr1**** | inositol 1,4,5-trisphosphate receptor 1 | 1.06 | 1.90E-05 |
|  | *Prkcg* | protein kinase C, gamma | 0.77 | 1.06E-03 |
|  | *Dlg4* | discs large MAGUK scaffold protein 4 | 0.71 | 7.9E-04 |

Note: The different markers in the same column represent significant difference at 0.001, 0.01 and 0.05 level, respectively. *** P<0.001, **P<0.01, *P<0.05. ^#^denotes genes with no changes in key candidate DETs validated by qPCR.
