# Supplementary material for: Effects of latent infection of Toxoplasma gondii strains with different genotypes on mouse behavior and brain transcripts
Source: Parasit Vectors. 2025 May 26;18:190. doi: 10.1186/s13071-025-06819-7 (PMC12107737; doi:10.1186/s13071-025-06819-7)
Supplement: Supplementary file 2 — Additional file 2: Table S2. Candidate key DETs corresponding to down-regulated genes in the brain tissue of Wh6 strain and LHG strain before and after infection. [file 13071_2025_6819_MOESM2_ESM.docx]

**Additional: Table S2** Candidate key DETs corresponding to down-regulated genes in the brain tissues of Wh6 strain and LHG strain before and after infection.

|  | **Gene Symbol** | **Full name of gene** | **Log_2_(FC)** | **P value** |
| --- | --- | --- | --- | --- |
| Wh6 | *Sept4** | septin 4 | -1.11 | 0.0018 |
|  | *Kcng4** | Potassium voltage-gated channel subfamily G member 4 | -1.08 | 0.0023 |
|  | *Unc13c*** | protein unc-13 homolog C | -1.01 | 4.37E-05 |
|  | *Doc2b*^#^ | double C2, beta | -0.98 | 3.51E-05 |
|  | *Arntl*^#^ | aryl hydrocarbon receptor nuclear translocator like | -0.95 | 0.0023 |
|  | *Slc17a6*^#^ | solute carrier family 17 (sodium-dependent inorganic phosphate cotransporter), member 6 | -0.94 | 3.90E-06 |
|  | *Fgf13* | fibroblast growth factor 13 | -0.93 | 0.00047 |
|  | *Ptch1*^#^ | patched 1 | -0.92 | 0.0040 |
|  | *Prkcg** | protein kinase C gamma type | -0.86 | 0.00055 |
|  | *Grin2c* | calcium channel, voltage-dependent, gamma subunit 2 | -0.82 | 0.0060 |
| LHG | *Ndrg2*** | N-myc downstream regulated gene 2 | -0.90 | 2.89E-06 |
|  | *Frzb*^#^ | frizzled-related protein | -0.88 | 6.36E-04 |
|  | *Crh** | corticotropin releasing hormone | -0.77 | 6.12E-03 |
|  | *Gng4*^#^ | guanine nucleotide binding protein (G protein), gamma 4 | -0.96 | 1.07E-04 |
| LHG vs Wh6 | *Ccl2** | C-C motif chemokine ligand 2 | -1.55 | 1.39E-10 |
|  | *Ccl7*^#^ | C-C motif chemokine ligand 7 | -1.35 | 2.55E-08 |
|  | *Nupr1** | nuclear protein transcription regulator 1 | -1.20 | 2.32E-06 |
|  | *H2-Q4** | histocompatibility 2, Q region locus 4 | -1.07 | 6.68E-07 |
|  | *Socs3*^#^ | suppressor of cytokine signaling 3 | -1.02 | 7.15E-05 |
|  | *H2-Aa*^#^ | histocompatibility 2, class II antigen A, alpha | -0.97 | 2.32E-05 |
|  | *Ifi209* | interferon activated gene 209 | -0.95 | 8.20E-04 |
|  | *Ccl12** | C-C motif chemokine ligand 12 | -0.92 | 1.10E-04 |
|  | *Slc38a3** | solute carrier family 38, member 3 | -0.91 | 1.39E-03 |
|  | *Sfrp1*^#^ | secreted frizzled-related protein 1 | -0.91 | 1.31E-03 |

Note: The different markers in the same column represent significant difference at 0.001, 0.01 and 0.05 level, respectively. *** P<0.001, **P<0.01, *P<0.05. ^#^denotes genes with no changes in key candidate DETs validated by qPCR.
